# Temperature-Dependent Structural Properties of Nickel and Cobalt Selenite Hydrates as Solar Water Evaporators

**DOI:** 10.3390/ma17112482

**Published:** 2024-05-21

**Authors:** Anastasiia Taranova, Kamran Akbar, Elisa Moretti, Alberto Vomiero, Giuseppe Pezzotti, Tatsuro Morita, Elia Marin, Wenliang Zhu

**Affiliations:** 1 Ceramic Physics Laboratory, Kyoto Institute of Technology, Sakyo-ku, Matsugasaki, Kyoto 606-8585, Japan; anastasiia.taranova@unive.it (A.T.); pezzotti@kit.ac.jp (G.P.); elia-marin@kit.ac.jp (E.M.); 2 Department of Molecular Sciences and Nanosystems, Ca’ Foscari University of Venice, Via Torino 155, 30172 Venezia Mestre, Italy; kamran.akbar@unive.it (K.A.); elisa.moretti@unive.it (E.M.); 3 Division of Materials Science, Department of Engineering Sciences and Mathematics, Luleå University of Technology, SE-971 87 Luleå, Sweden; 4 Faculty of Mechanical Engineering, Kyoto Institute of Technology, Sakyo-ku, Matsugasaki, Kyoto 606-8585, Japan; morita@kit.ac.jp

**Keywords:** nickel selenite hydrates, cobalt selenite hydrates, microstructure, thermal stability, solar water evaporation

## Abstract

Solar water evaporation offers a promising solution to address global water scarcity, utilizing renewable energy for purification and desalination. Transition-metal selenite hydrates (specifically nickel and cobalt) have shown potential as solar absorbers with high evaporation rates of 1.83 and 2.34 kg∙m^−2^∙h^−1^, but the reported discrepancy in evaporation rate deserves further investigation. This investigation aims to clarify their thermal stability for applications and determine the underlying mechanisms responsible for the differences. Nickel and cobalt selenite hydrate compositions were synthesized and investigated via thermogravimetric analysis, X-ray diffraction, and Raman spectroscopy to assess their temperature-induced structural and compositional variations. The results reveal distinct phase transitions and structural alterations under various temperature conditions for these two photothermal materials, providing valuable insights into the factors influencing water transportation and evaporation rates.

## 1. Introduction

Nowadays, there is an urgent problem arising from the continuous increase in global water scarcity [1,2,3]. Based on the United Nations report, over 2.3 billion people are facing significant water stress [4]. An essential solution capable of providing the required reliable life support is desalination—the process of extracting salt and minerals from seawater [5]. However, the pursuit of sustainable and economically feasible freshwater extraction methods has become paramount. Freshwater scarcity poses profound challenges, particularly in coastal regions where seawater is abundant. Traditional widespread desalination methods like reverse osmosis or distillation, dominant in arid regions such as the United Arab Emirates, Saudi Arabia, and Israel, pose significant drawbacks due to their substantial energy requirements, including both economic and environmental loads [6]. Investigating innovative approaches is crucial in addressing these challenges. Solar water evaporation emerges as a promising technology capable of purifying water and desalinating seawater [7]. Unlike conventional methods, solar water evaporation harnesses renewable energy from the sun, ensuring sustainability while minimizing greenhouse gas emissions [8].

Recent advancements in materials science have spurred the development of photothermal materials capable of efficiently harvesting the entire solar spectrum and converting it into heat [9]. The technology of interfacial solar evaporation involves placing photothermal materials at the water–air interface to directly convert solar energy into vapor [10]. To enhance efficiency, interfacial solar steam generation utilizes a porous thermal barrier with low thermal conductivity, effectively isolating solar absorbers from bulk water to minimize heat loss. Furthermore, porous structures or water channels serve as capillaries, facilitating water transport within solar absorbers. 

The efficiency of solar evaporation devices significantly depends on the solar absorber (photothermal materials), substrate (the material that provides water transport from bulk water to absorber), and thermal insulation. The request for photothermal materials that possess both excellent broad solar absorption and desirable hydrophilicity has long been a challenge in water purification technology. The organic-based structures exhibit promising properties post-carbonization and are known for their excellent hydrophilicity [11,12,13]. The current literature presents a noticeable gap in broad inorganic-based intrinsic solar absorbers with high hydrophilicity, making it difficult to find materials that seamlessly combine both attributes.

A promising route to address the global water crisis lies in the investigation of hydrates-containing compositions for solar-driven water evaporation [14,15]. Hydrate compounds possess crystal lattices that contain water molecules, making them suitable for application as solar water absorbers. Integrating the hydrates into solar water evaporators could offer an effective solution, harnessing the power of sunlight to facilitate water evaporation and purification, thus potentially alleviating water scarcity issues on a global scale. The combination of efficiency, affordability, and durability makes hydrates a compelling solution for sustainable water desalination. The efficiency of hydrate compositions is notable because they can absorb significant amounts of water molecules, which enhances the evaporation process with minimal solar energy input. The economic viability for large-scale deployment of hydrate compositions as absorbers is based on the low production costs, which not only maximize water output but also underscore the cost-effectiveness. Furthermore, the durability to resist harsh environmental conditions enhances their appeal for widespread implications, offering long-term solutions to water shortage challenges. 

Recently, we demonstrated that transition-metal selenite hydrates (earth-abundant metals Ni and Co) possess high solar absorbance (>96%) in the solar spectral range, as well as excellent hydrophilicity, which plays a key role in water transport in the solar steam generation [16]. The solar water evaporation rate of compositions deposited on the glass–wool membrane achieved 1.83 kg∙m^−2^∙h^−1^ and 2.34 kg∙m^−2^∙h^−1^ for nickel selenite hydrate and cobalt selenite hydrate, exceeding the theoretical limit of 1.47 kg∙m^−2^∙h^−1^ for 2D evaporators (see Supplementary Note S1) [17]. The hydrophilic properties observed in selenite hydrates arise from the presence of “water of hydration” trapped within its crystal lattice. 

Note that for practical applications of photothermal materials, the materials will be subjected to exposure to sun irradiation and experience a relatively high temperature (~30 °C –> 100 °C) [18,19,20]. In particular, in the case of dry conditions and/or continuous sun irradiation, a higher temperature will be obtained, which may induce further compositional/structural alterations to the materials. Accordingly, the clarification of the thermal stability of the constituent components and possible defect-related chemical reactions, as well as the examination of the applicable threshold temperature of the materials, will be crucial for applications. In this research, we attempted to investigate the intricate mechanisms governing water transportation and to uncover the factors responsible for the difference in evaporation rates between the two selenite hydrate photothermal materials. The thermal stability of the synthesized nickel and cobalt selenite hydrates was first analyzed by thermogravimetric analysis (TGA) to examine the possible compositional variation. Based on the observed compositional mass loss, a series of thermal treatments were performed at several different temperatures, and X-ray diffraction (XRD) and Raman spectroscopy were applied to assess their temperature-induced structural variations. The results reveal distinct temperature-dependent alterations of phase and structure for nickel and cobalt selenite hydrates, providing valuable insights into the factors influencing water transportation and evaporation rates.

## 2. Materials and Methods

### 2.1. Sample Preparation

The investigated nickel selenite hydrate and cobalt selenite hydrate samples (now referred to as NiSe and CoSe, hereafter) were prepared by aqueous synthesis [16]. First, 10 mM of nickel (II) acetate (or cobalt (II) acetate, Sigma-Aldrich, St. Louis, MO, USA) was dissolved in 50 mL of deionized water. Then, a NaHSe solution was prepared by dissolving 120 mM of NaBH_4_ (Sigma-Aldrich, St. Louis, MO, USA) in 50 mL of deionized water and adding 237 mg of Se powder (Sigma-Aldrich, St. Louis, MO, USA). The Ni(II) acetate (or Co(II) acetate) solution was heated to 100 °C in an oil bath and immediately mixed with the fresh NaHSe solution. The mixture was boiled in the oil bath for 180 min at 100 °C, cooled to room temperature, and washed with deionized water and ethanol several times to eliminate impurities. Finally, the products were dried at 70 °C overnight to become nanopowders. 

### 2.2. Characterizations

#### 2.2.1. Thermogravimetric Analysis

The thermal stability of the obtained NiSe and CoSe samples was evaluated using TGA equipment (Discovery TGA, TA Instruments, New Castle, DE, USA). TGA measurements were performed on approximately 50 mg samples placed on platinum pans. The samples were heated from room temperature (RT) to 800 °C at a rate of 10 °C∙min^−1^ in air. Moreover, additional experiments were also performed for the heating in the low-temperature region up to 40 °C and 70 °C, with a heating rate of 1 °C∙min^−1^ to avoid overheating and obtain more precise data. After reaching the setting temperature, the temperature was kept for 30 min for both cases. 

#### 2.2.2. Open Air Annealing

The samples were heated from room temperature to 100 °C, 200 °C, and 400 °C, respectively, at a rate of 10 °C∙min^−1^ in air and held up for 30 min (NHK-120BS-2, Nitto Kagaku Co., LTD., Nagoya, Japan).

#### 2.2.3. X-ray Diffraction Analysis

The crystalline structure of the thermally treated samples was determined by XRD using a Miniflex 600 diffractometer (Rigaku, Co., Tokyo, Japan) with an X-ray excitation (40 kV, 15 mA). Cu K_α_ radiation (wavelength of 1.5418 Å) was used to record the XRD patterns over the angular range from 10° to 70° with a step size of 0.05°. The evaluation of X-ray diffraction patterns was conducted using the MDI Jade 6.0 software. The peak position, intensity, and full width at half maximum (FWHM) of each phase were measured using LabSpec 5 software, and the intensity ratios were calculated after spectral deconvolution.

#### 2.2.4. Raman Spectroscopic Analysis

Raman measurements were performed via a Raman Touch (Nanophoton, Co., Osaka, Japan) equipped with Peltier-cooled CCD. The Raman spectra were collected using a green laser (an excitation wavelength of 532 nm) at 3.15 mW in XY Averaging measurement mode. The choice of green laser and low power is because of the high thermal sensitivity of the studied samples in order to reduce laser irradiation-induced damage to the surface of the materials.

## 3. Results and Discussion

**Figure 1:**
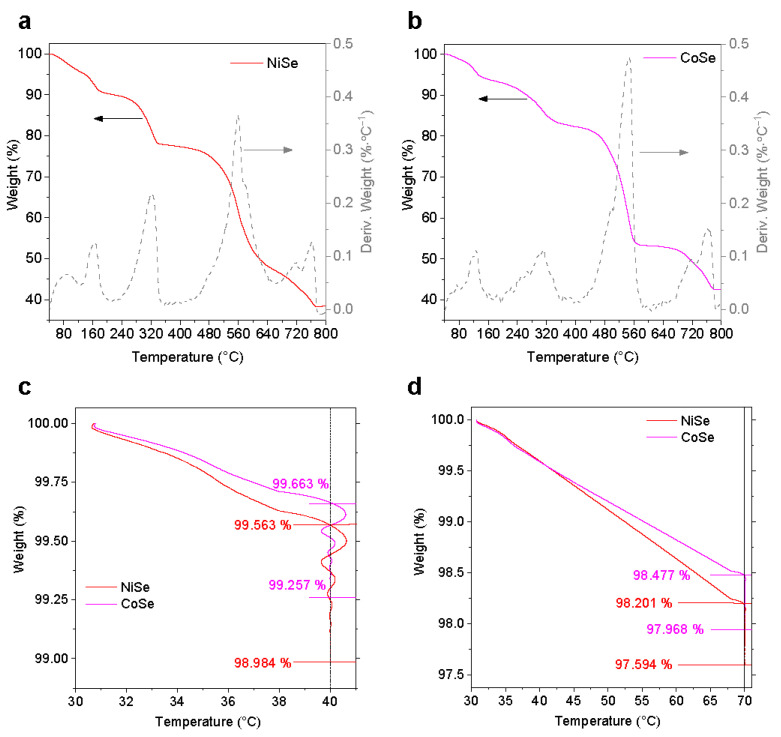
TGA and DTG thermograms for NiSe (**a**) and CoSe (**b**) up to 800 °C, and TGA curves of the two samples with holding time of 30 min at 40 °C (**c**) and 70 °C (**d**).

To understand the thermal stability of the samples, TGA analysis was first performed in a relatively large range from RT to 800 °C, as the results presented in Figure 1a,b, showing relatively similar thermograms of the decomposition process of NiSe and CoSe. To identify the number of decomposition steps, the derivative of each thermogram (DTG, %∙°C^−1^) is also presented. 

As can be seen from the profiles of the TG curves, both samples undergo several steps of mass reduction, as confirmed by the presence of different DTG peaks. In the low-temperature region, the mass of the samples shows a slight but gradual decrease with increasing temperature up to around 180 °C and 140 °C for NiSe and CoSe, respectively. DTG analysis reveals that the first and second steps are consecutive and overlapped: In the DTG curves, both NiSe and CoSe exhibit a peak at around 80 °C, while the positions of the second peak are different, located at around 160 °C and 120 °C, respectively. Increasing the temperature up to 330–360 °C results in a weight loss of 21.87% and 16.73% for NiSe and CoSe, respectively, along with the appearance of a strong DTG peak at 320 °C. At 800 °C, a total weight loss of 61.52% and 57.38% can be detected for NiSe and CoSe, respectively. In the temperature range between 440 and 620 °C, the most significant mass reduction in the TG curves and the appearance of the most intense DTG peak can be observed, which indicates a weight loss of around 30% in this area, probably owing to the formation of NiSeO_3_ and CoSeO_3_ [21,22].

As can also be seen from Figure 1c,d, CoSe showed better stability than NiSe during both low-temperature treatments. The weight loss of CoSe during heating from 30 to 40 °C was estimated to be 0.337 %, and after 30 min, the final loss achieved 0.743 %, while the weight loss of NiSe amounted to 1.016%. A similar trend occurred at 70 °C, the CoSe powder lost 2.032% of weight, while NiSe lost 2.406%. The possible chemical and physical transformations that cause the observed decomposition steps will be discussed later.

**Figure 2:**
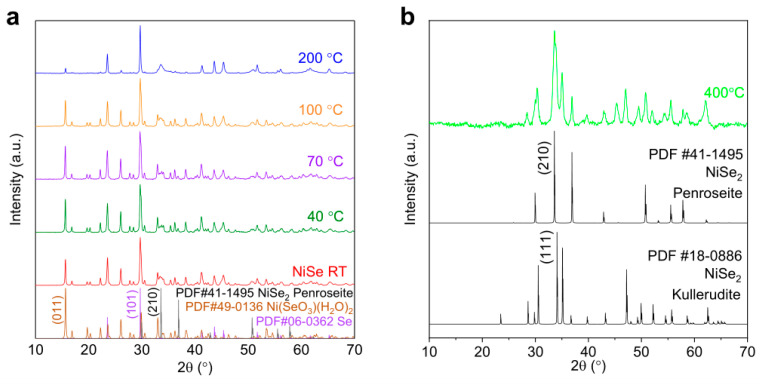
XRD patterns of NiSe at RT and annealed at temperatures up to 200 °C (**a**) and 400 °C (**b**). The PDF cards of the relevant phases are reported in (**a**,**b**).

Further analyses of the compositional/structural alteration of the samples induced by thermal treatments were conducted by XRD. Figure 2 depicts a comparison of the XRD patterns acquired from freshly synthesized NiSe and heat-treated samples. According to the XRD spectrum for the untreated NiSe powder obtained from the aqueous synthesis at RT (Figure 2a), the most prominent peaks are observed at 2*θ* ≈ 29.71° and 15.64°, which mainly correspond to the (101) plane of the selenium Se phase (PDF #06-0362), and the (011) plane of Ni(SeO_3_)(H_2_O)_2_ (PDF#49-0136), respectively. The predominant phase within the sample is Ni(SeO_3_)(H_2_O)_2_, notwithstanding the observation that the peak at 2*θ* ≈ 29.71° exhibits the highest intensity. This heightened intensity arises from the overlapping of the peaks originating from both the Ni(SeO_3_)(H_2_O)_2_ phase and elemental Se, resulting in an overall enhanced peak intensity. Furthermore, a minor fraction of NiSe_2_ in the Penroseite structure (PDF#41-1495) might also exist, marked by the primary peak at 2*θ* ≈ 33.41°. From the data presented in Figure 2, the phase composition of the heat-treated NiSe powders remains barely altered at temperatures ≤ 100 °C. This observation underscores the thermal stability of the crystalline phases within the NiSe material under these relatively low-temperature conditions. At an elevated temperature of 200 °C, the primary Se phase and the minor NiSe_2_ component exhibited consistent phase characteristics, indicating their stability under these conditions. Concurrently, a notable reduction in the intensity of the Ni(SeO_3_)(H_2_O)_2_ peaks was observed, signifying an indicative measure of the reduction in the hydrated phase and reflecting a thermally induced transformation or decomposition of Ni(SeO_3_)(H_2_O)_2_. This is consistent with the presence of the peak at 160 °C in the DTG curve (Figure 1a). As depicted in Figure 2b, upon thermal treatment at 400 °C for 30 min, a pronounced alteration in the phase and structural constitution of NiSe becomes evident. The NiSe sample treated at 400 °C is characterized by the coexistence of NiSe_2_ in two distinct structural configurations, Penroseite (PDF#41-1495) and Kullerudite (PDF#18-0886), with approximately equimolar proportions in its composition. This transformation highlights the substantial thermally induced metamorphosis in the crystalline phases and structural attributes of NiSe. In addition, no more signals from the elemental Se phase can be observed, as the melting point of Se is 221 °C. Therefore, both the decomposition of dehydrated Ni(SeO_3_)(H_2_O)_2_ and the evaporation/reaction of Se can contribute to the observed mass loss in the range from 200 to 360 °C and the appearance of the DTG peak at 320 °C shown in Figure 1a. Annealing of the samples at higher temperatures (>400 °C) may result in a further loss of Se and the formation of nonstoichimetric NiSe*_x_* phases, responsible for the mass reduction in the TG curve (Figure 1a). 

Initially, the XRD profiles of the specimens subjected to low-temperature treatments (T ≤ 100 °C) displayed a resemblance, necessitating a more intricate examination of the peaks associated with distinct phases using LabSpec 5 software to perform spectral deconvolution of the specific peaks. Note that the position and width of an XRD peak are associated with the crystalline structure and crystallinity, as well as the presence of defects and nonstoichiometric alterations to distort the lattice of the material. The selection of specific 2*θ* values for peak analysis was motivated by the imperative to mitigate potential overlaps among multiple phases and the aspiration to discern these peaks distinctly, thus enhancing the precision and quality of the analytical outcomes. 

**Figure 3:**
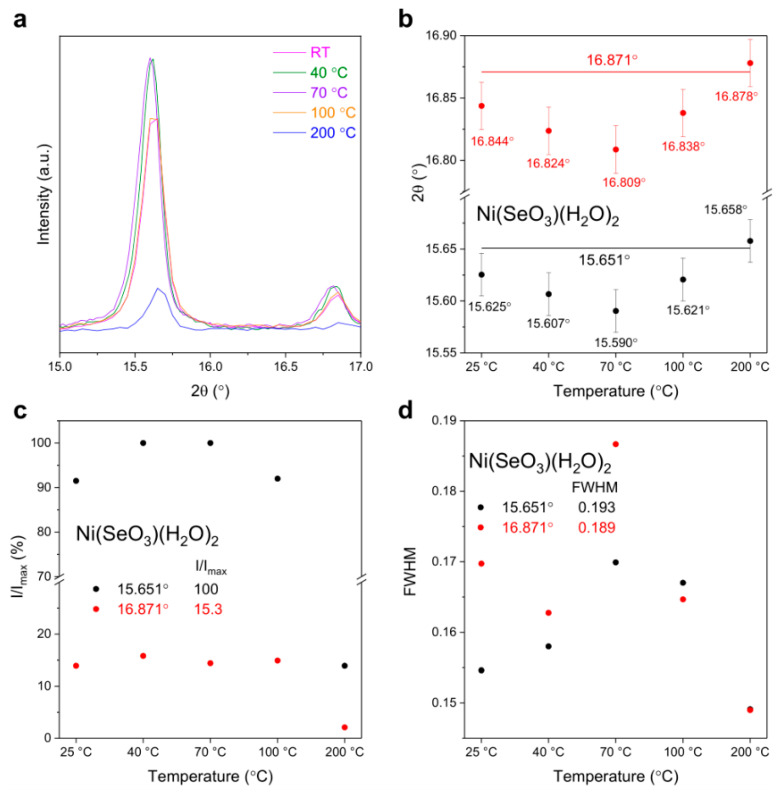
Comparison of the selected peaks representative for NiSeO_3_(H_2_O)_2_ (**a**) and variations in position (**b**), relative intensity (**c**), and FWHM (**d**) with temperature.

Figure 3a shows the related spectra of the selected peaks representative for NiSeO_3_(H_2_O)_2_ (15.6° and 16.8°, (011) and (10-1) planes, respectively), and Figure 3b–d show a comprehensive presentation of spectral deconvolution results, specifically providing detailed data regarding the position, relative intensity, and full width at half maximum as a function of temperature, taking advantages of the selected peaks. These data points provide a thorough and precise depiction of the distinctive characteristics of different heating temperatures. 

Note that in the graph for the peak position (Figure 3b), the lines provided represent the 2*θ* position of the peak from the database, and the error bar was estimated based on several XRD measurements of each powder. As the temperature increases from RT to 70 °C, the selected 2*θ* peaks show a gradual reduction to lower values but then an increase as the temperature further rises up to 200 °C. In the meanwhile, the FWHM values first increase to 70 °C then decrease at higher temperatures. It is worth noting that in this study, two distinct peaks originating from the same components were used for confirmation, and the observed similar trend of variations in the plot demonstrates the validity of the obtained results. Concerning the intensity variation, the strongest peak at 15.6° of NiSeO_3_(H_2_O)_2_ for the sample thermally treated at 70 °C was used as a normalization factor (the maximum intensity) for comparisons. In general, the relative intensity of NiSeO_3_(H_2_O)_2_ also shows a first increase to 70 °C and then a decrease as temperature increases (Figure 3c). With respect to the strong peak at 15.6° that corresponds to the (011) plane, the peak at 16.9° shows a relatively small intensity variation, which might indicate a slight structural alteration of NiSeO_3_(H_2_O)_2_ upon thermal treatment. 

In general, the as-prepared NiSe sample may have a certain amount of adsorbed water on the particle surfaces because of the hydrophilicity and porous nature of the material, besides the presence of hydrated water in the particles. Consequently, upon thermal treatment or sun irradiation, the increase in surface temperature results in the process of desorption and evaporation of the adsorbed water at relatively lower temperatures. That is to say, at lower temperatures, typically below 100 °C, the material undergoes a remarkable transformation in its surface lattice structure attributed to the removal of surface-bound water molecules, leading to dehydration. This can be supported by the observation of a mass reduction in the TGA curve and the presence of the DTG peak at around 80 °C (cf. Figure 1a). The dehydration process causes a peak shift and an increase in FWHM of NiSeO_3_(H_2_O)_2_.

As the temperature continues to rise, a distinct shift occurs in the dominant process. At higher temperatures, the removal of internally bound hydrated water becomes increasingly prevalent (cf. strong DTG peak at around 160 °C in Figure 1a). This transition triggers an intriguing internal transportation of hydrated water within the material. The removal of the hydrated water from the lattice in NiSeO_3_(H_2_O)_2_ can increase the structural order in NiSeO_3_, and thus, a higher crystallinity can be expected, showing a decrease in the FWHM of the diffraction peak at higher temperatures. In addition, the 2*θ* position will shift to higher values due to a dehydration-induced structural contraction.

**Figure 4:**
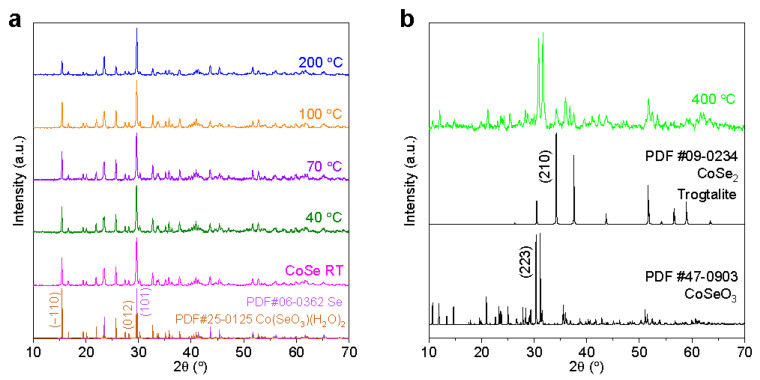
XRD patterns of CoSe at RT and annealed at temperatures up to 200 °C (**a**) and annealed at 400 °C (**b**). The PDF cards of the relevant phases are reported in (**a**,**b**).

Similarly, in the case of untreated CoSe, as illustrated by the XRD spectrum of CoSe RT in Figure 4a, noteworthy peaks manifest at 2*θ* ≈ 29.71°, corresponding to the (101) plane of the selenium Se phase (PDF#06-0362) and at 2*θ* ≈ 29.61° attributed to the (012) plane of Co(SeO_3_)(H_2_O)_2_ phase (PDF#25-0125). The heightened intensity of the strongest sample peak arises from the superposition of signals stemming from these two phases. Figure 4a also presents data demonstrating the stability of the phase composition of heat-treated CoSe powders, with negligible variations observed at temperatures <200 °C and a minor reduction in Co(SeO_3_)(H_2_O)_2_ content at 200 °C. Upon elevating the temperature to 400 °C, as shown in Figure 4b, substantial phase transformations take place as the signals from Se and Co(SeO_3_)(H_2_O)_2_ disappear. The primary phase is now identified as CoSeO_3_ (PDF#47-0903), marked by a dominant peak at 2*θ* ≈ 31.65°, and CoSe_2_ in the Trogtalite structure (PDF#09-0234), characterized by a principal peak at 2*θ* ≈ 37.62°. This alteration underscores the significant annealing-induced shift in the crystalline phases within the CoSe material at temperatures higher than 200 °C. 

Comparing the XRD results of CoSe and NiSe, the presence of a significant amount of Co(SeO_3_)(H_2_O)_2_ at 200 °C (cf. strong peak at 15.6° in Figure 4a) indicates less dehydration of the hydrated water in CoSe. The formation of CoSe_2_ from Co(SeO_3_)(H_2_O)_2_ appears only in the range 200–400 °C and the structure of oxyselenide is kept even at 400 °C, while the NiSe sample annealed at 400 °C has completely converted to NiSe_2_. The difference in the behavior of phase transition between NiSe and CoSe under identical reaction environments can be explained by different energy barriers. For the transition from Co^2+^ (Co(SeO_3_)(H_2_O)_2_) to Co^4+^ (CoSe_2_), the standard reduction potential reaches +3.34 V, and for Se^0,^ it is a challenge to capture two electrons from Co^2+^. The reduction potential in the case of Ni^2+^ → Ni^4+^ is only +1.59 V, which explains the easier formation of NiSe_2_ [23].

**Figure 5:**
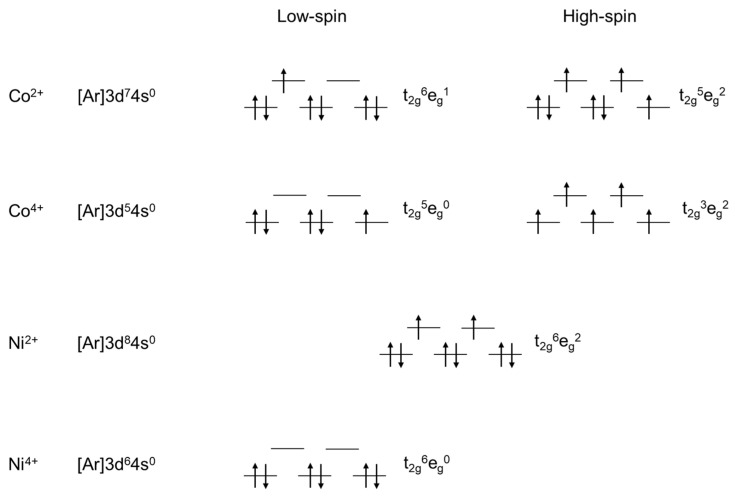
Electronic configuration of nickel and cobalt ions. An arrow pointing upwards indicates one spin direction, while a downward pointing arrow indicates the other direction.

The formation of NiSe_2_ in NiSe and the suppression of CoSe_2_ in CoSe can be described based on ligand field theory. This theory suggests that ligands can be perceived as negative charges arranged in specific geometric patterns—octahedral or tetrahedral. In an octahedral crystal field, the 3*d* orbitals of the transition metal ion are split into two groups, with the energy difference described by the Δoct crystal-field splitting parameter: t_2g_ orbital symmetry with 0.4Δoct, and e_g_ orbital symmetry with 0.6Δoct. Figure 5 shows an illustration of the 3*d* orbital configuration of nickel and cobalt ions in selenite hydrate complex: Co^2+^ and Ni^2+^ have spin states with 3*d* orbital configuration t_2g_^6^e_g_^1^ and t_2g_^6^e_g_^2^, respectively. In NiSe_2,_ the Ni^4+^ owns the 3*d* orbital configuration as t_2g_^6^e_g_^0^ because Ni^2+^ is easier to donate 2 electrons from the e_g_ orbitals for becoming NiSe_2_ rather than for Co^2+^ for the formation of CoSe_2_, which needs additional energy to break the electron pairs in the t_2g_ orbitals. Therefore, the way for the oxidation of Ni^2+^ to Ni^4+^ is more favorable than that of cobalt. In addition, the presence of asymmetrically occupied *d*-orbitals (e_g_^1^) in Co(SeO_3_)(H_2_O)_2_ may cause a distortion of the octahedral structure due to the Jahn-Teller effect. This structural alteration is assumed to partly stabilize or destabilize the hydrated water along different directions in CoSe, so the structural asymmetry may result in the appearance of two peaks at 120 and 250 °C in the DTG curve of CoSe (Figure 1b), with respect to the single DTG peak at 160 °C for NiSe. Accordingly, although a relatively minor fraction of dehydration occurs upon annealing at 200 °C, the mobility of hydrated water in CoSe can be more efficient in certain directions (cf. the lower DTG peak position).

**Figure 6:**
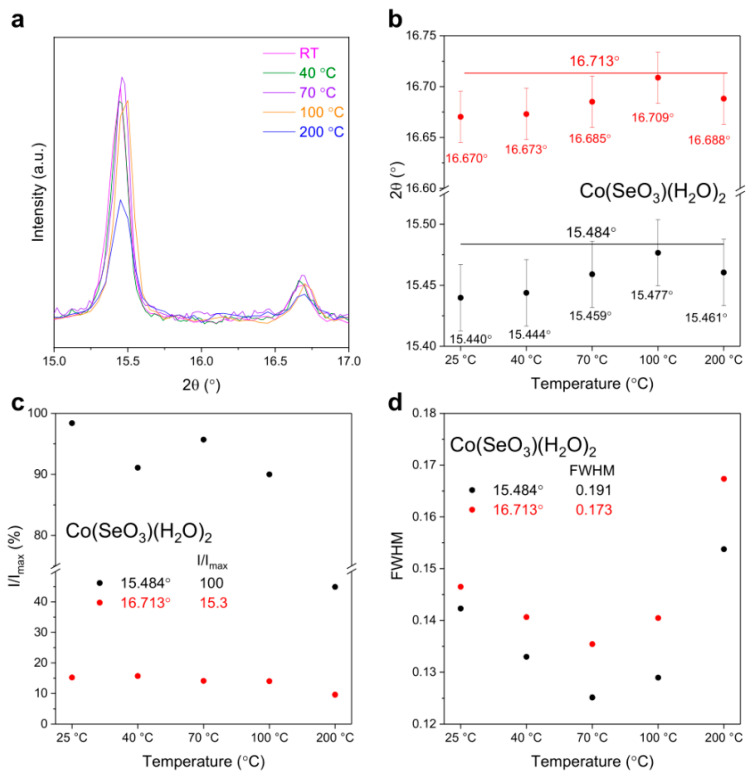
Comparison of the selected peaks representative for CoSeO_3_(H_2_O)_2_ (**a**) and variations in position (**b**), relative intensity (**c**), and FWHM (**d**) with temperature.

Figure 6 provides more XRD results pertaining to the CoSe sample, specifically, the enlarged spectra, the 2*θ* position, relative intensity, and FWHM for Co(SeO_3_)(H_2_O)_2_. At temperatures below 200 °C, unlike NiSe, with increasing temperature, the 2*θ* peaks slightly shift to higher values along with a reduction in FWHM, indicating a surface lattice reconstruction. In addition, the peak at 16.7° (−101 plane) shows an insignificant variation in the relative intensity compared with the strong peak at 15.5° (−110 plane). This might be owing to the competition between the desorption of surface water and the movement/dehydration of hydrated water in certain crystallographic directions induced by asymmetric lattice distortions and might also be associated with the capability of internal hydrated water transport within the material at low temperatures. At higher temperatures (200 °C), although Co(SeO_3_)(H_2_O)_2_ exhibits greater stability, retaining its structural integrity, the movement of internal hydrated water along different directions becomes the predominant process, which might cause a higher degree of structural disorder and result in the observed peak broadening. 

**Figure 7:**
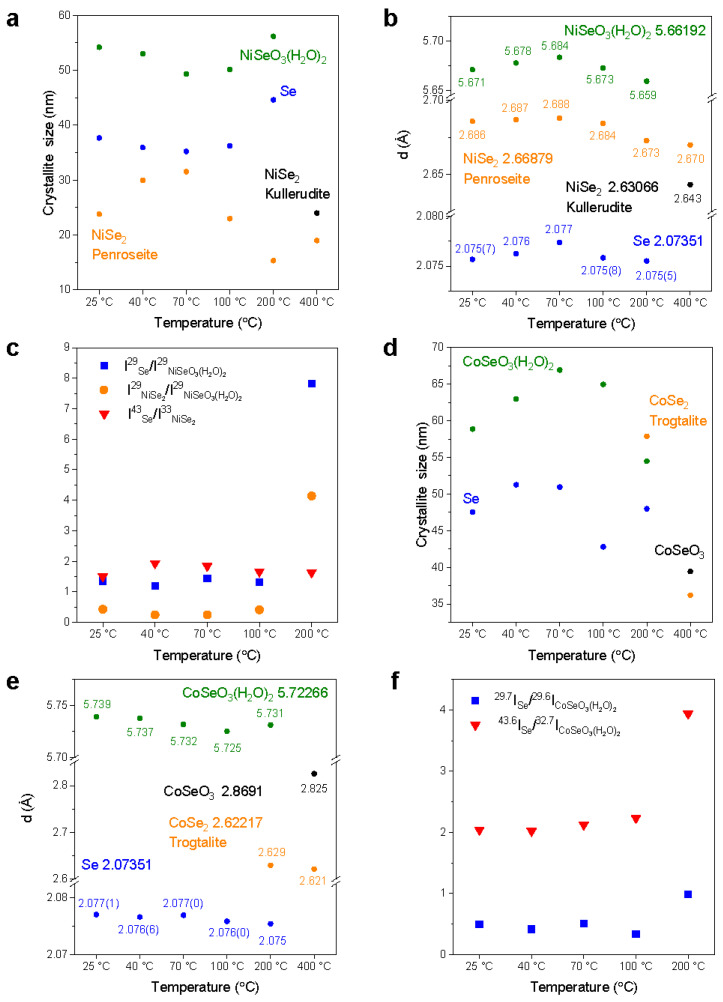
Temperature-dependent crystallite size, *d*-spacing, and variations in the intensity ratios, *I*(Se)/*I*(Ni(SeO_3_)(H_2_O)_2_), and *I*(Se)/*I*(Co(SeO_3_)(H_2_O)_2_) for NiSe (**a**–**c**) and CoSe (**d**–**f**).

Figure 7 shows the variations in the crystallite size and the spacing of the crystal layers *d* with temperature for Ni(SeO_3_)(H_2_O)_2_ and Co(SeO_3_)(H_2_O)_2_. The determination of crystallite (grain) size *D* was conducted by applying the Scherrer equation as outlined in Equation (1) [24]:

(1) D=Kλ/βcosθ

where *K* is the numerical factor (Scherrer constant), *λ* is the CuK_α_ wavelength (0.154056 nm), and *β* is the full width at half maximum (in radians) of the (hkl) peaks at the diffraction angle 2*θ*. The FWHM of the diffraction peak was calculated by spectral fitting using a Gaussian function. *K* is a numerical factor referred to as the crystallite-shape factor. Its value is contingent on the crystallite shape and the definitions of the average crystallite size and width. Within the range of 0.87–1.0, *K*’s numerical value can vary significantly, although it is commonly accepted as 0.9 [25].

And the spacing of the crystal layers *d* (Å) was calculated via Bragg’s law (Equation (2)) [26]:

(2) nλ=2dsinθ

where *n* is the diffraction order (1, 2, 3…), *λ* is the wavelength (Å), and *θ* is the glancing angle (radian).

With increasing temperature <100 °C, Ni(SeO_3_)(H_2_O)_2_ shows a reduction in the crystallite size owing to the removal/dehydration of surface water, while Co(SeO_3_)(H_2_O)_2_ presents a lattice expansion (reduction in *d*) owing to the movement of internal hydrated water in certain crystallographic directions. At higher temperatures, the occurrence of dehydration/movement of internal bound hydrated water Ni(SeO_3_)(H_2_O)_2_ may result in a lattice expansion, while Co(SeO_3_)(H_2_O)_2_ presents a lattice reduction due to the competition of the dehydration of the internally bound hydrated water. Figure 7c,f show a comparison of the intensity ratios of the peaks of the components for NiSe and CoSe in response to the change in annealing temperature. As can be seen, the NiSe sample shows a slight increase in the ratio, *I*(Se)/*I*(Ni(SeO_3_)(H_2_O)_2_) in the low-temperature region (<100 °C), and then a significant increase at 200 °C, while for Co(SeO_3_)(H_2_O)_2_, the intensity ratio barely changes at low temperatures, and then slightly increases at 200 °C, confirming minor dehydration. 

**Figure 8:**
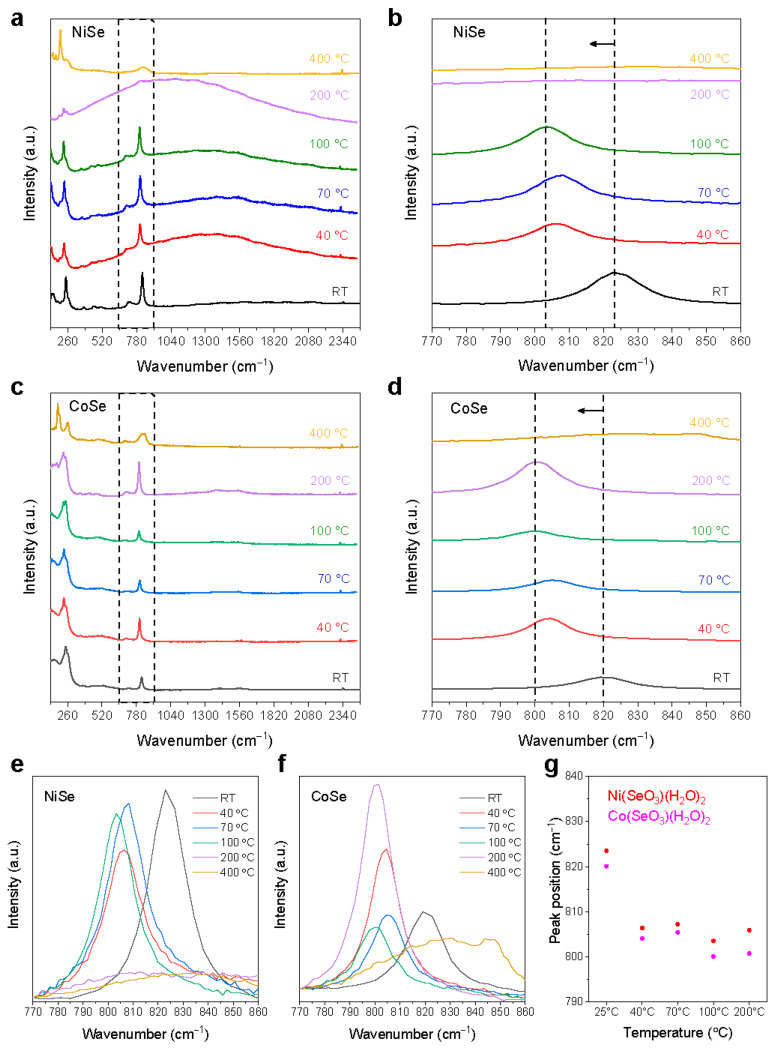
Temperature-dependent Raman spectra and enlarged plots of the selected spectral region 770–860 cm^−1^ of NiSe (**a**–**c**) and CoSe (**d**–**f**), peak position at around 820 cm^−1^ vs. temperature (**g**). An arrow pointing to a shift of the peak position.

To further examine the annealing-induced structural alterations, micro-Raman spectroscopic analysis was performed on the samples, as their typical Raman spectra are shown in Figure 8. For untreated NiSe and CoSe samples, the Raman spectra exhibit similar profiles with the presence of two main peaks despite a difference in peak intensities (Figure 8a,d). At RT, one main peak originated from the symmetric stretching of (SeO_3_)^2−^ in NiSeO_3_(H_2_O)_2_ can be seen at about 823.5 cm^−1^ and at 819.8 cm^−1^ for CoSe [27,28]. In NiSe, the low-intensive peak of the Se-Se stretching mode is observed at 217 cm^−1^, which was close to the value observed in other diselenides [29,30]. The stretching of the Se–Se bond in metallic Se is associated with the second main peak at around 244.9 cm^−1^ for NiSe and at 244.8 cm^−1^ for CoSe [31,32].

Upon heating, the spectral morphology shows a slight change in the low-temperature range for both samples, while a marked decrease in the intensity of the Raman bands was found for NiSe but not for CoSe upon annealing at 200 °C. Further annealing at 400 °C results in the appearance of Raman signals from NiSe_2_ (~203 cm^−1^) or CoSe_2_ and Co(SeO_3_) (~185 and 201 cm^−1^) and the disappearance of the signals of Se and Ni(SeO_3_)(H_2_O)_2_ (Co(SeO_3_)(H_2_O)_2_), in good agreement with the XRD result. Figure 8b,d–f show an enlargement of the variation in the SeO_3_^2−^ peak at around 820 cm^−1^ for the samples. As can be seen, a clear shift of the peak position toward lower wavenumbers after annealing could be observed (Figure 8g), which is taken to be associated with the surface dehydration/desorption in NiSe and also the internal movement of hydrated water in CoSe. These Raman spectroscopic observations are in line with the XRD results. The perturbations of XRD and Raman spectra not only underscore the thermally induced changes in composition but also provide further insights into the structural alterations associated with temperature variations, offering a nuanced understanding of the dynamic behavior of NiSe and CoSe under thermal conditions. 

## 4. Conclusions

The study presents a multidisciplinary approach that combines the power of thermogravimetric analyses with advanced structural characterizations by XRD and Raman spectroscopy to understand the thermal stability and different photothermal behaviors of nickel and cobalt selenite hydrate materials. Both materials exhibit dynamic structural changes in response to temperature variations, showing different steps of mass loss due to stoichiometric/nonstoichiometric alterations and phase transitions. At low temperatures (below 100 °C), the occurrence of surface water dehydration (represented by a slight mass loss along with a TGA peak at 80 °C) induces a surface lattice reconstruction of the particles, resulting in a red shift of the Raman band of the selenite hydrate, as well as a slight variation in the XRD peak profiles. Owing to the difference in ligand fields, the asymmetric lattice distortions in CoSeO_3_(H_2_O)_2_ might alter the stabilization energy of the internally bound hydrated water, partly stabilizing or destabilizing it along different directions, thus resulting in the splitting of the DTG peak associated with its dehydration upon thermal treatments in CoSe. Accordingly, the higher mobility of the hydrated water in certain directions in Co(SeO_3_)(H_2_O)_2_ (cf. a lower DTG peak position) can be more efficient for water transport upon sun irradiation or heating, despite a relatively minor fraction of dehydration upon annealing at 200 °C because of the retention of the stabilized hydrated water in cobalt selenite hydrate (cf. DTG peak at 250 °C). The competition between the desorption of surface adsorbed water and the movement/dehydration of hydrated water within the material results in distinct temperature-dependent variations in the XRD peaks of NiSeO_3_(H_2_O)_2_ and Co(SeO_3_)(H_2_O)_2_ at low temperatures. At high temperatures (>200 °C), the materials may experience further removals of internal hydrated water, reaction/evaporation of elemental metallic Se, and transformation to NiSe_2_ or CoSe_2_, as well as to nonstoichiometric NiSe*_x_* (CoSe*_x_*) at higher temperatures. Because of the smaller energy barrier of Ni^2+^ for oxidation and the asymmetric structural distortion of CoSeO_3_(H_2_O)_2_, CoSe shows a relatively higher thermal stability with respect to NiSe. However, during applications of the solar water evaporators, the decrease in energy requirements for water transportation of the internally bound hydrated water may lead to a higher evaporation rate and, as a result, to the effective solar water evaporation.

## Data Availability

The original contributions presented in the study are included in the article/Supplementary Material, further inquiries can be directed to the corresponding authors.

## Supplementary Materials

The following supporting information can be downloaded at: https://www.mdpi.com/article/10.3390/ma17112482/s1.
