# Supplementary material for: Temperature-Dependent Structural Properties of Nickel and Cobalt Selenite Hydrates as Solar Water Evaporators
Source: Materials (Basel). 2024 May 21;17(11):2482. doi: 10.3390/ma17112482 (PMC11173136; doi:10.3390/ma17112482)
Supplement: Supplementary file 1 [file materials-17-02482-s001.zip › materials-2964278-supplementary.pdf]

Note S1

The theoretical limit of vapor output was calculated assuming a 100 % solar-to-vapor energy transfer efficiency by using the equation:

$$Theoretical\ limit = \frac{P_{in}}{H_v - H_w}$$

where P_{in} – the incident solar energy on the surface of the generator in 1 hour;

H_v – the specific enthalpies of vapor;

H_w – the specific enthalpies of water.

Because the surface temperature of the generator is uniform, the qualitative temperature for determining the specific enthalpy of the vapor and water is complex. For the convenience of the calculations, it is assumed that the evaporation process proceeded from 20 °C water to 20 °C vapor. Therefore, the theoretical limits under 25 $mW \cdot cm^{-2}$, 50 $mW \cdot cm^{-2}$, 100 $mW \cdot cm^{-2}$, and 120 $mW \cdot cm^{-2}$ illumination were 0.37 $L \cdot m^{-2} \cdot h^{-1}$, 0.73 $L \cdot m^{-2} \cdot h^{-1}$, 1.47 $L \cdot m^{-2} \cdot h^{-1}$, and 1.76 $L \cdot m^{-2} \cdot h^{-1}$, respectively. For the detailed experiments, the relative error introduced by deviations in the temperatures of the vapor and water from 20 °C was less than ±4 % [1].

References

1. Li, X.; Li, J.; Lu, J.; Xu, N.; Chen, C.; Min, X.; Zhu, B.; Li, H.; Zhou, L.; Zhu, S.; et al. Enhancement of Interfacial Solar Vapor Generation by Environmental Energy. *Joule* **2018**, *2*, 1331–1338, doi:10.1016/j.joule.2018.04.004.
